# Suicide by flecainide: a case report and review of the literature

**DOI:** 10.1007/s12024-026-01188-8

**Published:** 2026-03-07

**Authors:** Angelo Montana, Fabrizio Lo Faro, Chiara Turchi, Federica Alessandrini, Arianna Giorgetti

**Affiliations:** 1 Department of Biomedical Sciences and Public Health, Università Politecnica Delle Marche, 60126 Ancona, Italy; 2 Unit of Legal Medicine, Department of Medical and Surgical Sciences, University of Bologna, Via Irnerio 49, 40126 Bologna, Italy

**Keywords:** Forensic pathology, Forensic toxicology, Anti-arrhythmic drug, Intoxication, Suicide

## Abstract

**Purpose:**

Flecainide is a prescription drug used to treat or prevent various types of arrhythmias by decreasing conduction velocity. Despite its therapeutic utility, flecainide has a narrow safety margin and potential for fatal toxicity. This study presents a forensic case of fatal flecainide intoxication and investigates the role of pharmacogenetics in drug metabolism and toxicity.

**Methods:**

A 27-year-old medical doctor, prescribed flecainide, was found deceased. Postmortem examination included histological and toxicological analyses of blood and urine samples, with flecainide quantification by a validated method. CYP2D6 and CYP3A5 were genotyped to check for variants of the respective drug-metabolizing enzymes. A literature review of fatal and non-fatal flecainide intoxication is also presented.

**Results:**

Flecainide concentrations measured 3.411 mg/l and 2.962 mg/l in central and peripheral blood, respectively. Pharmacogenetic analysis identified a CYP3A5 *3/*3 poor metabolizer genotype, likely contributing to reduced drug clearance and increased toxicity. Histology revealed mild coronary stenosis and signs consistent with cardiogenic shock. The death was deemed a suicide, possibly facilitated by abnormal metabolic phenotype in a mildly compromised cardiac substrate.

**Conclusions:**

This case highlights the complexities in diagnosing lethal flecainide intoxication, particularly considering postmortem concentration variability and individual metabolic differences. Integrating pharmacogenetic data enhances interpretation of toxicological findings and may guide personalized therapeutic strategies to mitigate risk. Further research is warranted to clarify genetic influences on flecainide toxicity.

**Supplementary Information:**

The online version contains supplementary material available at https://doi.org/10.1007/s12024-026-01188-8.

## Introduction

Flecainide acetate (N-(2-piperidinylmethyl)-2,5-bis(2,2,2-trifluoroethoxy)benzamide monoacetate) is a class IC antiarrhythmic drug first synthesized in 1972 [1]. It is used to treat or prevent various types of potentially life-threatening cardiac arrhythmias, including paroxysmal supraventricular tachycardia (PSVT) and atrial fibrillation/atrial flutter, in patients without structural heart disease [2–5]. Flecainide interacts with several cardiac ion channels, blocking fast-inward sodium (Na^+^) channels, especially in the His-Purkinje system. The drug has also been proposed to inhibit the cardiac ryanodine receptor (RyR2), which is implicated in calcium (Ca^2+^) release and reuptake between the cytosol and the intracellular sarcoplasmic reticulum (SR), leading to decreased conduction velocity and prolonged ventricular refractoriness [6, 7].

Following oral administration, flecainide is promptly absorbed from the gastrointestinal tract. Peak plasma concentration is reached within three hours after administration, with high oral bioavailability (approximately 90%) [3]. The drug has a relatively long plasma half-life, low binding to human plasma proteins (mean 40%), and exhibits a wide volume of distribution throughout the body (8–9 l/kg) [8, 9]. Flecainide is primarily metabolized in the liver via the cytochrome P450 (CYP) system, particularly by CYP2D6 to meta-O-dealkylated flecainide or its lactam derivative [6]. Meta-O-dealkylated flecainide retains about 20% of flecainide’s activity [9]. Both the metabolites and the parent drug are typically excreted in urine [10, 11].

Flecainide has a narrow therapeutic index, with therapeutic plasma concentrations around 0.2–0.8 mg/l [8]. Adverse reactions have been reported at levels ≥ 0.7–1 mg/l, with a steep dose–response curve, especially when structural heart disease is present [12–14]. The drug can cause new or worsened arrhythmias, such as ventricular tachycardia or torsades de pointes, due to proarrhythmic effects, but it may also induce a negative inotropic effect with sinus bradycardia and atrioventricular block. Other adverse effects include edema, drowsiness, diplopia, headache, tremors, confusion, stupor, convulsions, and death [12, 15].

From a clinical perspective, treating flecainide overdose poses challenges due to its high bioavailability and lack of specific antidotes. In cases of severe manifestations, the administration of high-dose sodium bicarbonate should be considered, along with intensive care procedures, including support of adequate oxygenation, ventilation, and cardiovascular function [16, 17].

In forensic pathology, flecainide is not typically included among the drugs routinely tested for suspected fatal intoxications, and it is rarely encountered as a cause of death. Moreover, interpreting postmortem results might be challenging due to postmortem acidification, which might increase levels in cardiac blood [18].

Here we present a case of fatal flecainide intoxication in a young medical doctor, including data on central and peripheral blood levels, along with postmortem pharmacogenetic analysis.

A literature review on fatal and non-fatal intoxications involving flecainide is also presented.

## Case Description

A 27-year-old medical doctor was found unconscious in his vehicle, parked near his workplace by his mother. She had been alerted by a phone call from him during which he claimed that his health was deteriorating—a statement that may have referred to acute adverse effects possibly related to his flecainide intake.

Emergency medical services were promptly notified; however, resuscitation efforts were unsuccessful.

A thorough investigation of the death scene was conducted by law enforcement officers in collaboration with a forensic pathologist. The examination was initially performed at the vehicle but did not yield any notable findings. Following the recommendations of the forensic pathologist, the investigation was extended to include the victim’s workplace. This led to the discovery of several empty packs of flecainide in his office; each package had originally contained 20 tablets of 100 mg each. As circumstantial information, it was reported that on the day preceding his death, an altercation occurred between him and his girlfriend. Consequently, he spent the night at his parents' residence. The following morning, he self-prescribed two packs of flecainide tablets.

## Materials and methods

### Postmortem examination

A complete postmortem examination was performed 2 days after death and included external examination, internal sectioning, and collection of fluids and biological material for histological, toxicological, and genetic analyses. During the autopsy, the whole heart was collected and then fixed in 10% buffered formalin. For histological analysis, tissue specimens were dehydrated in graded ethanol, cleared in xylene, and embedded in paraffin. The obtained paraffin blocks were cut (4 μm thickness) using a microtome; sections were mounted on silane-coated slides (Dako, Glostrup, Denmark) and stored at room temperature. Sections were stained with H&E and observed using a Zeiss Axioplan light microscope (Carl Zeiss, Oberkochen, Germany). Subsequently, images were obtained using a Zeiss AxioCam MRc5 digital camera (Carl Zeiss, Oberkochen, Germany).

### Reference materials and chemicals

Reference standards of flecainide and proadifen hydrochloride (SKF 525A), used as the internal standard (IS), were purchased from Cayman Chemical (Michigan, USA) and stored at -20 °C until analysis. HPLC-grade methanol, dichloromethane, acetonitrile (ACN), isopropanol, and water were supplied by Carlo Erba (Cornaredo, Italy). Formic acid was obtained from Carl Roth GmbH (Karlsruhe, Germany). Mobile phases consisted of 0.1% formic acid in water (A) and ACN (B) and were freshly prepared prior to analysis.

### Toxicological analyses

Toxicological analyses were conducted on blood and urine samples collected during the postmortem examination. The following drugs of abuse were screened in urine using the DRI-CEDIA immunoassay (Thermo Fisher Scientific): amphetamines (cut-off 500 ng/ml), benzodiazepines (cut-off 300 ng/ml), cannabinoids (cut-off 50 ng/ml), cocaine (cut-off 300 ng/ml), ecstasy (cut-off 500 ng/ml), methadone (cut-off 100 ng/ml) and opiates (cut-off 300 ng/ml).

Toxic volatile organic compounds, including ethyl ether, chloroform, benzene, methyl alcohol, and ethyl alcohol (with a lower limit of quantification of 0.04 ng/mL) were analysed in cardiac blood diluted 1:1 with distilled water; 0.5 ml of IS 0.1% was added, and the samples were placed into vials for analysis. A volume of 0.3 ml of the headspace was analysed using an Agilent Technologies® 6850 gas chromatograph (Palo Alto; CA, USA) equipped with a flame ionization detector (FID), an automatic sampling device (HS Gerstel MPS 2) and an HP-B Alc gas chromatographic column (7.5 m, 0.32 mm i.d., 0.2 µm film thickness). The operating conditions were as follows: injector temperature 250 °C; split ratio 1:20; detector temperature 300 °C; fuel gas hydrogen at 30 ml/min; combustion gas air at 300 ml/min; carrier gas hydrogen at 2 ml/min; make-up nitrogen at 30 ml/min.

#### Sample preparation for flecainide analysis

Briefly, ten microlitres of 50 ng/μl IS (SKF 525A), 0.5 ml of 2 N NaOH and 5 ml of a 9:1 (v/v) dichloromethane/isopropanol mixture were added to 100 μl of central and peripheral blood. The tubes were stirred using a roller mixer for 30 min and centrifuged at 5,589 × g for 15 min.

Supernatants were transferred into clean tubes and dried under nitrogen at 40–50 °C for 30 min. Samples were reconstituted in 100 μl of methanol and transferred into autosampler vials prior to injection of 5 μl into the chromatographic system.

#### High-performance liquid chromatography-tandem mass spectrometry (HPLC–MS/MS) analysis

Flecainide concentrations were determined by high-performance liquid chromatography coupled with tandem mass spectrometry (HPLC–MS/MS). To this scope, a previously validated method for forensic purposes, developed in accordance with the recommendations of the Organization of Scientific Area Committees (OSAC) for Forensic Science [19], was used. To obtain concentrations within the calibration range, blood samples were analyzed after 1:2 and 1:3 dilutions in deionized-distilled water. Details of the previous validation are reported in the Supplementary Material.

An aliquot of 5 µl was injected into an Agilent 1290 Infinity HPLC system (Agilent Technologies, Palo Alto, CA, USA) interfaced with an Agilent 6470A triple quadrupole equipped with an electrospray ionization (ESI) source operating in positive mode. Data were acquired using MassHunter® Workstation Quantitative Analysis Software (version 10.0, Agilent Technologies, Palo Alto, CA, USA).

Separation was performed on a Kinetexs® 2.6 µm Phenyl-Hexyl column from Phenomenex® (100 mm × 2.1 mm). The initial conditions were 5% B, held for 1 min, increased gradually to 50% B within 2 min, increased to 95% B within 4 min, then decreased to 5% B and held until 6 min. The autosampler and column oven temperatures were 10 °C and 37 °C, respectively. Run time was 6 min, with a flow rate of 0.4 ml/min. Multiple reaction monitoring (MRM) analysis was performed in positive mode for the following transitions: *m/z* 415.0 397.9 and 301.1 for flecainide; *m/z* 389.9 241 and m/z 222 for SKF-525A.

#### Validation of method

The limit of detection (LOD), lower limit of quantification (LLOQ), linearity, analytical bias, carry-over, matrix effect (ME), recovery (RE), and dilution integrity were evaluated throughout the method validation process in accordance with the recommendations of the Organization of Scientific Area Committees (OSAC) for Forensic Science [18]. The method was validated over five consecutive days. The best fit-calibration model was a linear least-squares regression model with 1/x^2^ weighting, as confirmed by Mandel test coefficients (p value and Fcrit 95%) [19].

Satisfactory linearity within the concentration range of 0.0005–1 mg/l (R^2^ > 0.99) was observed. In agreement with the established international criteria, accuracy and precision were within ± 20% at the LLOQ and within ± 15% at all the QC samples.

### Pharmacogenetics testing

Human genomic DNA was extracted using the QIAamp® DSP DNA Mini Kit (Qiagen) according to the manufacturer’s protocol. Genotyping was performed by Massively Parallel Sequencing (MPS) technology using the Ion AmpliSeq™ Pharmacogenomics Panel (Thermo Fisher Scientific), a single-pool panel containing 119 primer pairs used for the library preparation from genomic “hot spot” regions that play a critical role in drug-metabolizing enzymes (DMEs). It amplifies 136 polymorphic regions in 40 genes. The analysis was focused on CYP2D6 and CYP3A5. More details on the pharmacogenetic testing are reported in the Supplementary Material.

Libraries were prepared according to the Ion AmpliSeq™ Library Kit 2.0 protocol (Life Technologies, Thermo Fisher Scientific, MAN0006735 rev C.0). Libraries enrichment and chip loading steps were performed using the Ion Chef™ (TFS) and the Ion 520™ Kit. Sequencing was performed on the Ion Gene Studio S5™ (TFS). All raw data were processed by the Torrent Suite (v. 5.12.3) and the reads aligned against the human reference genome (GRCh37/hg19). Coverage analysis was carried out by the Coverage Analysis (v5.12.0.0) plugin, which provided information on mapped reads, on-target percentage, and mean depth of coverage downloadable for each sample library. Allelic calls for each library were performed using the Variant Caller Plugin (v.5.12.0.4). The Integrative Genomics Viewer (IGV) software was used to verify the variant calls. Haplotype translation files were created using data from PharmGKB and used in conjunction with AlleleTyper™ Software (ThermoFisher Scientific) to determine star-allele results.

### Literature review

A comprehensive literature search on fatal and non-fatal intoxications involving flecainide, with critical review of the collected studies, was performed. An electronic search of PubMed, Science Direct, Scopus, Google Scholar, and the Excerpta Medica Database (EMBASE) was conducted from database inception to December 2023. Database-specific search features with truncations (represented by an asterisk in this article) and multiple keywords (represented by quotation marks in this article) were employed. The search terms, used alone or in combination, included: flecainide, fatal*, abuse, death, toxic*, poisoning, intoxication, adverse effects, autopsy, and suicide.

The references of all papers were examined and cross-referenced to further identify relevant literature. The data collection process included study selection and data extraction. The following inclusion criteria were used: (1) original research articles, (2) reviews and mini-reviews, (3) case reports and case series, (4) papers written in English, (5) detection of flecainide in blood or serum or plasma, alone or in combination with other substances, (6) acute intoxications due to flecainide, (7) deaths due to flecainide intake.

Two reviewers processed the articles independently. The titles and abstracts were reviewed in a first phase, followed by a second phase consisting of evaluation of the full text of each potentially relevant study, reconsidering criteria 1–7. Through consultation and debate with a third reviewer, any divergence regarding study eligibility was resolved.

After inclusion, the following data were extracted from each case: gender and age of the victim/patient; flecainide concentrations determined in blood, serum, or plasma and in additional matrices, when available; other drugs detected in blood; manner and cause of death or intoxication, with particular reference to flecainide consumption, whether deemed intentional or not, and the involvement of additional drugs; main clinical, circumstantial, and/or postmortem findings.

## Results

### Case report

**Fig. 1:**
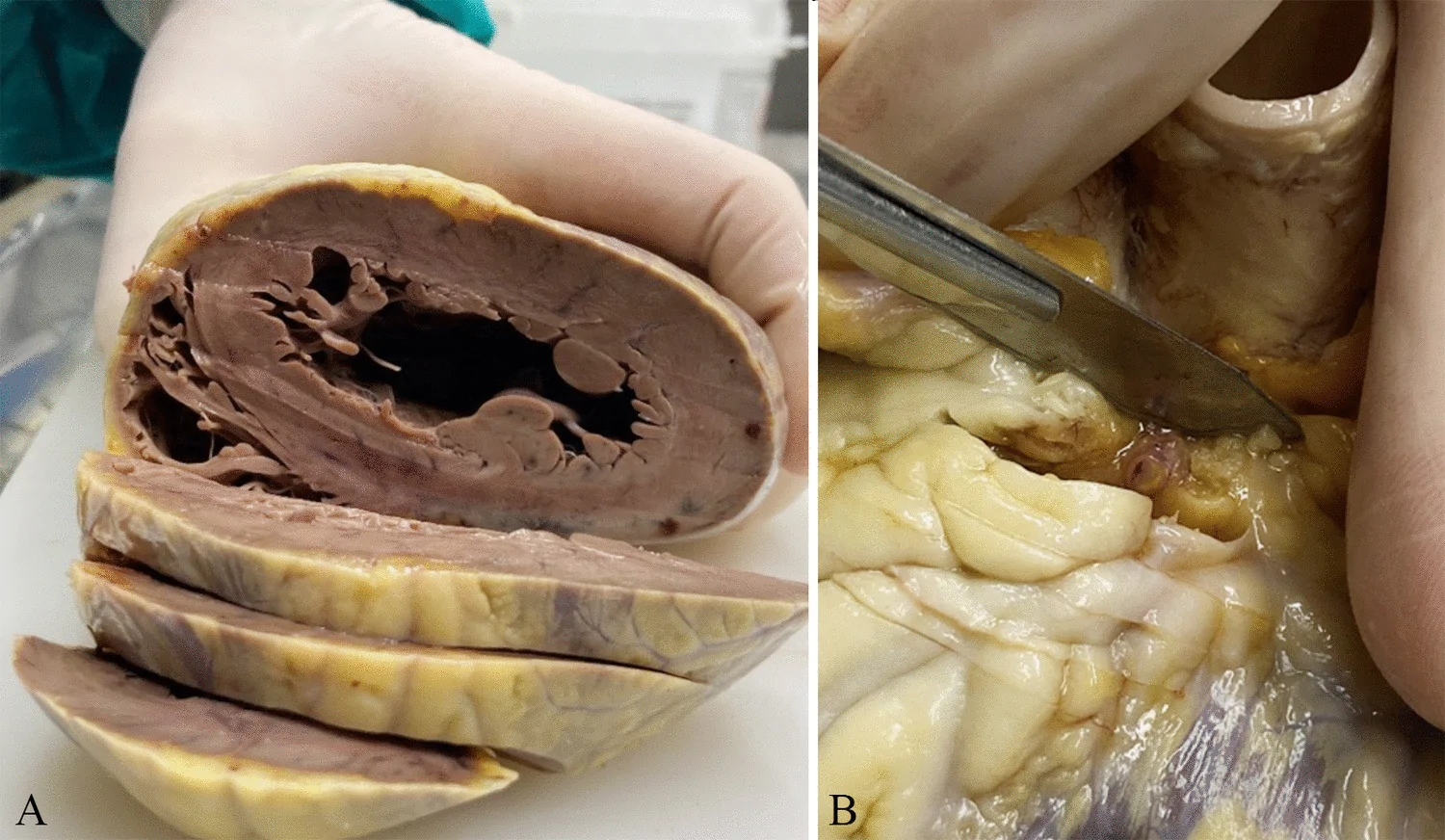
In **A**, the heart after formaldehyde fixation showing ventricular chambers with normal thicknesses. In **B**, the stenosis of the anterior interventricular branch

The external examination of the body (height: 165 cm, weight: 81 kg) revealed no signs of external trauma. Hypostasis was observed on the upper half of the body, including the head, neck, superior thorax, and upper limbs. The autopsy revealed brain (brain weight: 1,320 g) and pulmonary edema (lung weight: 950 g) showing congestion and petechiae with abundant foam upon sectioning. The heart (weight: 375 g) had a regular shape (12 × 9.5 cm). Myocardial thickness measurements were within the upper limits of the normal range (anterior septum: 1.4 cm; anterior wall of the left ventricle: 1.5 cm; lateral wall of the left ventricle: 1.5 cm; posterior wall of the left ventricle: 1 cm; lateral wall of the right ventricle: 0.5 cm) (Fig. 1a).

A moderate stenosis was found in the anterior interventricular branch of the left coronary artery at approximately 2 cm from its origin (Fig. 1b), while the other branches showed only mild to moderate coronary sclerosis. The myocardium and valvular apparatus appeared normal. Other organs exhibited signs consistent with diffuse visceral congestion.

Histological analysis confirmed brain perineuronal and perivascular edema, as well as massive endoalveolar edema in the lungs, together with liver and visceral congestion.

**Fig. 2:**
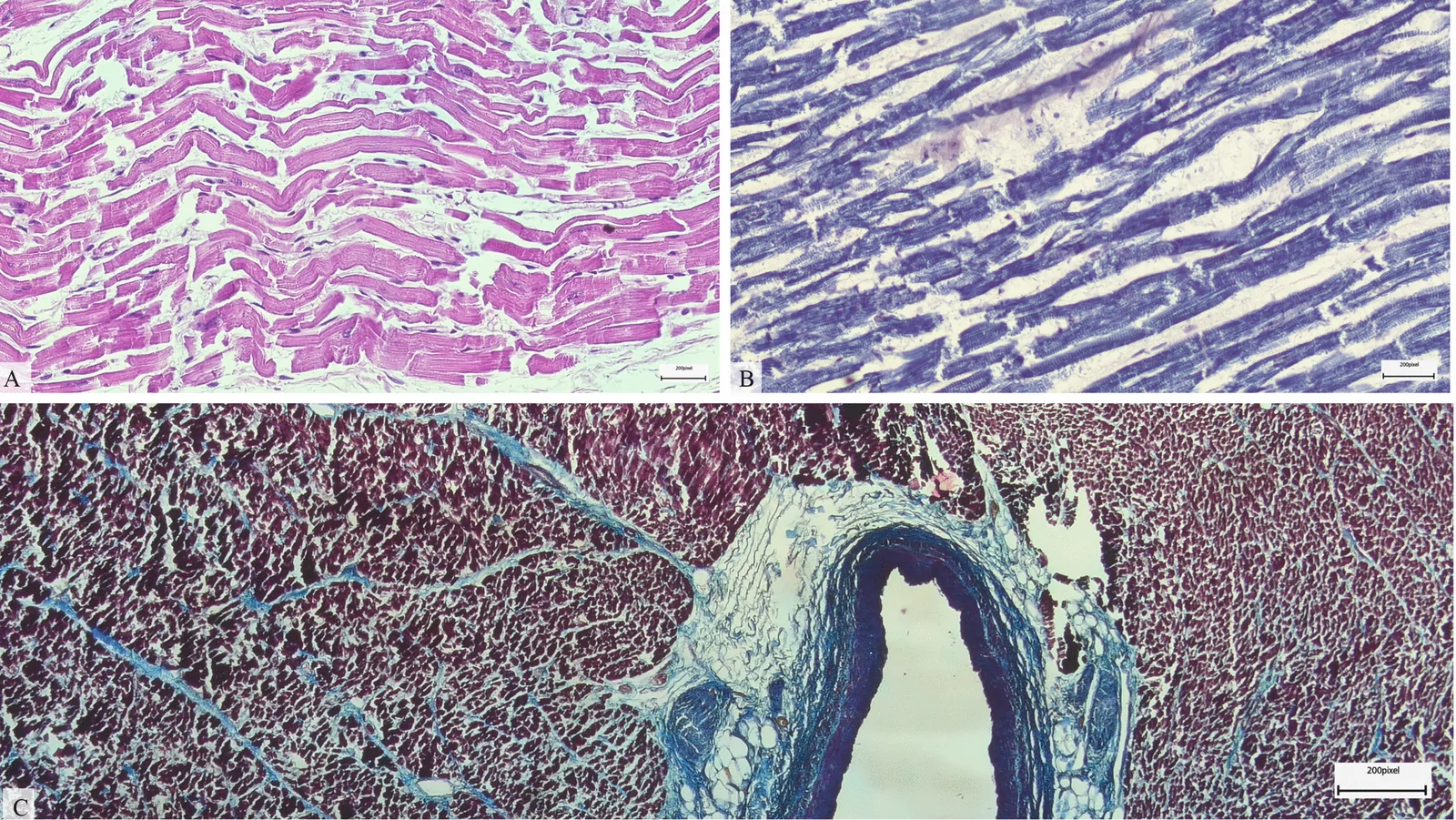
Microscopy of the heart. **A**: hematoxylin–eosin (H&E) × 40, myofiber waviness; **B**: PTAH × 80, PTAH staining showing hypereosinophilia and coagulative necrosis of cardiomyocytes, with an enlargement within the red square; **C**: Transverse histological section of the anteroseptal regions of left ventricle showing focal replacement fibrosis (Heidenhain trichrome o.m. × 8)

Specimens from the heart and coronary arteries, additionally stained with Heidenhain’s Azan trichrome (Fig. 2) revealed several features: myofiber waviness and disarray (a), foci of hypereosinophilia and coagulative necrosis of cardiomyocytes (b), interstitial edema (c). The anteroseptal regions of the left ventricle were characterized by localized replacement fibrosis (d).

**Fig. 3:**
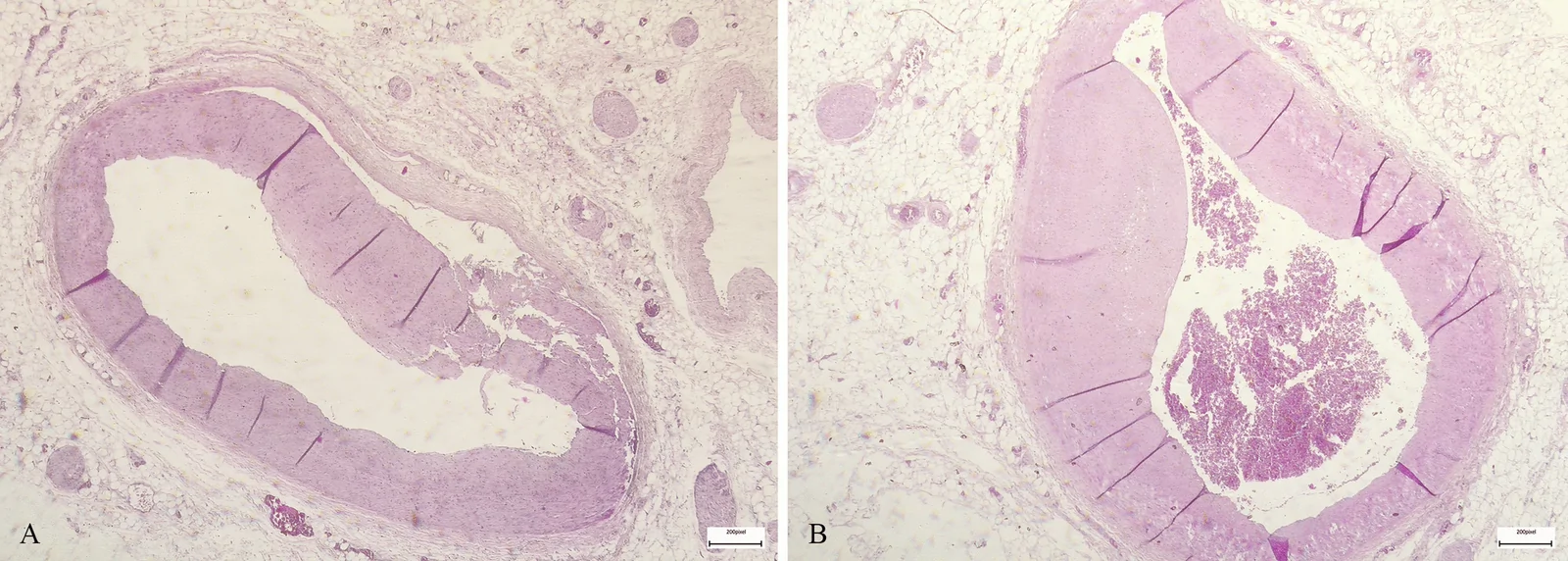
Descending coronary artery, showing mild atherosclerosis in **A** and moderate non-obstructive stenosis in **B**

All coronary arteries displayed diffuse intimal and medial thickening. Specifically, moderate-grade stenosis was noted in the proximal and intermediate portions of the left anterior descending coronary artery (Fig. 3).

LC–MS/MS analysis allowed the identification of flecainide in central (3.411 mg/l) and peripheral blood (2.962 mg/l). Other substances, including ethanol, were not detected in the analyzed biological matrices.

Pharmacogenetic testing for variants in cytochrome P450 enzymes, particularly CYP2D6 and CYP3A5 [11, 20–22], was additionally performed on a blood sample collected during autopsy. The AlleleTyper™ Software was not able to determine the star alleles for the CYP2D6 gene. Results of CYP2D6 genotyping are shown in the Supplementary Material Section C. The CYP3A5 gene was homozygous for the *3 allele: (CYP3A5*3/*3), which is associated with increased systemic exposure (AUC) and lower oral clearance of flecainide in healthy individuals, indicating a poor metabolizer phenotype.

Regarding CYP2D6, the primary enzyme responsible for flecainide metabolism, approximately 3.4% of individuals in the Italian population are classified as poor metabolizers (PMs), while around 35% are intermediate metabolizers (IMs). The remainder are primarily extensive metabolizers (EMs, 35%), with a smaller proportion identified as ultrarapid metabolizers (UMs) [23].

Clinical and pharmacokinetic studies—primarily conducted in Asian populations—have demonstrated that CYP2D6 polymorphisms significantly reduce flecainide clearance and increase systemic drug exposure in IMs and PMs. In a Japanese pharmacokinetic study, flecainide oral clearance was found to be progressively lower across CYP2D6 genotypes: approximately 89% in EM/IM, 84% in EM/PM, 79% in IM/IM, and 73% in IM/PM individuals, compared with the EM/EM reference group [24].

In the European population, the CYP3A5*3 allele—a non-functional (PM) variant—is highly prevalent, with an estimated allele frequency of about 92%, meaning that CYP3A5*3/*3 homozygous PMs represent the vast majority. For instance, in a Sardinian cohort, the frequency of the PM phenotype was approximately 78.8%, with 19.3% IMs and only 1% NMs, reflecting similar trends across the Italian population [25].

A pharmacokinetic study conducted in healthy Chinese volunteers evaluated the effects of polymorphisms in CYP2D6, CYP3A5, CYP1A2, and ABCB1 on flecainide pharmacokinetics. Subjects homozygous for CYP3A5*3/*3 had a 26% higher AUC and 17% lower oral clearance compared with carriers of at least one functional *1 allele (*1/*1 or *1/*3) (p < 0.05). Interestingly, in this cohort, the CYP2D6 genotype did not significantly influence pharmacokinetics, suggesting that CYP3A5 may play a relevant secondary role in flecainide metabolism [26].

### Literature review

Case reports of fatal and non-fatal acute intoxications following flecainide intake are described in Table 1 [10, 22–33] and Supplementary Table [15, 34–56], respectively. Fifteen cases of fatal intoxication were identified in the literature.

**Table 1:** Results of literature review including cases of fatalities related to flecainide

Gender, Age (y–o)	Flecanide concentrations (mg/l or mg/kg)	Other drugs detected in blood/urine (mg/l or mg/kg for drugs, g/l for alcohol)	Manner/cause of death	Main clinical/circumstantial findings	Autopsy findings	Ref
F, 30	Antemortem whole blood: 2.01	Ondansetron	- Flecainide and ondansetron intoxication	History of peripartum cardiomyopathy, PE, and bipolar disorder. Brought to the ED during a cardiac arrest. Over the next 2 h the patient went in a wide-complex arrhythmia with prolonged QTc. Anoxic brain injury with care withdrawn	-	Gaylor JM. et al. 2019 [27]
F, 58	Cardiac blood: 10.16 Peripheral blood: 8.64 Liver 59.6 Bile 128 Brain: 4.19	-	Intentional flecainide intoxication	The patient had taken a box of 20 Almarytm® 100 mg tablets. Brought unconscious to the hospital and sent to the ICU with a diagnosis of abuse of antidysrhythmic, serious cardio circulatory insufficiency	Light atherosclerotic damage of the coronary arteries, in a morphologically normal heart	Vezzoli S. et al. 2017 [28]
F, 40	Plasma: 3.26	Betaxolol	Intentional flecainide intoxication	GCS 6 at admittance. Developed a broad complex bradycardia with type 1 Brugada pattern. Severe global hypokinesia followed by 3 cardiac arrests, cardiogenic shock and brain death after extracorporeal life support	-	Vivien B. et al. 2010 [29]
M, 78	Antemortem plasma: 2.5 Postmortem: Right cardiac blood 13.8 Left cardiac blood 44.2	-	Accidental flecainide intoxication	History of pacemaker implantation due to sick sinus syndrome, with flecainide prescription for 13 y. For 8 days before death, he took 400 mg/day of flecainide in the hospital	-	Yoshitome K. et al. 2008 [30]
F, 15	Subclavian blood:18.73 Urine: 28.3 Bile 23.8 Liver: 24.6 Stomach content 133.6 V.H: 15.42	Blood Ethanol 0.14 Carbamazepine:0.99 Tramadol: 0.32 Ndesalkilflurazepam: 0.07 Urine: Carbamazepine:0.09 Tramadol: 0.52 Ndesalkilflurazepam: 0.38	Intentional flecainide intoxication	History of depression and suicidal behavior. She was admitted to the hospital after flurazepam ingestion. Abdominal complaints. Further inquiry revealed that the victim had stolen flecainide pills in the hospital	Brain edema (1330 g). Lung edema (880 g) with pleural effusion. Vomit particles in the trachea. Tardieu spots on pericardium. Vascular congestion. At histology, pulmonary congestion and hemorrhagic edema, increased number of eosinophilic cells. A few myocytes with basophilic degeneration, some leucocytic sludging in the veins	Benijts T. et al. 2003 [10]
Case 1) M, 69 Case 2) M, 56	Case 1) Femoral blood: 5.4 Stomach content: 238 mg Case 2) Femoral blood: 1.2	Case 1) Diazepam:0.4 Ethanol: 0.8 Case 2) Satolol 0.37 Ethanol: 0.2	Case 1) Intentional flecainide intoxication Case 2) - Cardiac failure	Case 1) found dead in his residence after consuming excessive amounts of prescribed medication Case 2) history of hypertrophic obstructive cardiomyopathy treated with a variety of anti-arrhythmic agents. Taken to the hospital for cardiac arrest, unsuccessful treatment	Case 1) unremarkable Case 2) bilateral pulmonary congestion and edema. The heart showed disproportionate septal hypertrophy	Lynch MJ. et al. 2001 [31]
M, 65	Femoral blood: 7.7 Bile: 0.26 Liver: 18 Cerebrospinal fluid 0.17 Brain cortex: 0.22 Urine: 28.9 Gastric content: 43 mg	Ethanol: Femoral blood 1.75, codeine Urine: codeine, guaifenesin, clozapine, diclofenac metabolite	Intentional flecainide intoxication	History of chronic bronchitis. Found dead in his residence after consuming excessive amounts of flecainide prescribed for arrhythmia	Cardiac hypertrophy (heart weight 630 g, left ventricular wall thickness 1.8 cm), non-obstructive atherosclerotic lesions, nonspecific pulmonary congestion, hepatic steatosis	Romain N. et al. 1999 [32]
M, 36	Serum: 3.32	-	Intentional flecainide intoxication	He presented to the ED accident after deliberate overdose of 100 mg flecainide acetate tablets six hours previously. Died 12.5 h after hospital admission	-	Brazil E. et al. 1998 [33]
F, 20	Serum/plasma: 5.45	Ethanol	Intentional flecainide intoxication	No history of cardiovascular disorders. Agonal rhythm and no effective blood pressure. Started a CBS. Effective cardiac rhythm and blood pressure turned. The patient died 4 days after discharge for severe neurologic damage	-	Yasui RK. et al. 1996 [34]
M, 23	Femoral blood: 7.3 Urine 117 Stomach contents 19 mg Liver: 302	Ethanol Femoral blood 1.07 Urine 1.3 V.U: 1.13	Intentional flecainide intoxication	-	-	Sadler DW. et al 1995 [35]
Case 1) F, 33 Case 2) M, 15	Case 1) Blood:93.7 Urine: 82.4 Bile: 418.9 Liver: 550 Case 2) Blood: 100	Case 1) blood: ethanol 1.8 alprazolam < 0.001 µg/l; Case 2): -	Intentional flecainide intoxication	Case 1) history of depression; at the scene a suicide note and a bottle of flecainide (100 mg) were found Case 2) the decedent was not doing as well in school as his siblings; he took their mothers’ medications	Unremarkable	Rogers C. et al. 1993 [36]
M, 18	Blood 16.3 Liver 111 Stomach content 4260	-	Intentional flecainide intoxication	History of Marfan's syndrome treated with flecainide, 100 mg × 3, for recurrent episodes of ventricular tachycardia	Unremarkable	Forrest AR. et al. 1991 [37]
F, 22	Blood:13 Bile: 160 Urine: 54 V.H.:7.4 Liver: 180 Kidney: 74 Stomach contents 120	-	- Flecainide intoxication	History of palpitations, syncope, dizziness, frequent PVCs, and possible mitral valve prolapse disease. Treated with flecainide, 100 mg × 2; replaced with propranolol three months before death. Found unresponsive, treated for synus tachycardia and released. Experienced gastrointestinal distress, chest pain, bradycardia with hypotension	Unremarkable	Levine B. et al. 1990 [38]

CBS, cardiopulmonary bypass support; ED, emergency department; F, female; GCS: Glasgow coma scale; ICU, intensive care unit; M, male; PE, pulmonary embolism; PVCs, premature ventricular contractions; QTc, heart-rate corrected QT interval.—= not reported

The cases were almost evenly distributed between men (53.3%) and women (46.7%), with an age range of 15 to 78 years. The mean age was 38.5 years with a standard deviation (SD) of 21.3, and the median age was 33 years, with an interquartile range (IQR) of 20–58. The main cause of death was flecainide toxicity. Antemortem concentrations in fatal cases, obtained from plasma or serum, ranged from 2.0 mg/l to 5.4 mg/l, with a mean of 3.5 mg/l (SD = 3.5) and a median of 3.3 mg/l (IQR = 2.3–4.9). Postmortem concentrations were difficult to compare due to different or unspecified sampling sites. Notwithstanding this limitation, flecainide levels ranged from 1.2 mg/l to 100 mg/l, with a mean of 28.7 mg/l (SD = 35.5) and a median of 13.0 mg/l (IQR = 7.3–44.2). The substance was also found in urine, gastric content, cerebrospinal fluid, bile, vitreous humor, and in the liver, brain, and kidney tissues. Eleven of the 15 cases were classified as intentional intoxications, accounting for 73.3% of the total. In 8 cases (53.3%), flecainide was consumed in combination with psychotropic substances, such as ethanol, benzodiazepines, opioids, and/or prescription drugs. In most cases, death was preceded by wide-complex arrhythmias with prolonged QTc, atrial fibrillation, absence of effective blood pressure, cardiac arrest, and multiple organ failure. In 7 cases (46.7%), postmortem findings were deemed unremarkable and/or the heart was morphologically normal. Atherosclerotic damage and cardiac hypertrophy were observed in 2 cases (13.3%).

A total of 26 cases of non-fatal intoxication were identified (see Supplementary Table D). Again, women and men were almost evenly distributed, with a slight predominance of women (53.8%). The age of the patients ranged from 3 days to 86 years, with a mean of 46.3 years (SD = 20.9) and a median of 47.0 years (IQR = 28–57), excluding paediatric cases. In 16 cases, flecainide was ingested alone, while in 10 cases it was consumed in combination with drugs of abuse (such as alcohol and benzodiazepines) or prescription drugs. For adult intoxications, serum or plasma flecainide concentrations ranged from 1.39 mg/l to 1940 mg/l.

Among adult or adolescent cases, intentional ingestion of flecainide was reported in 16 of 22 (72.7%) cases.

Common symptoms included prolonged PR or QTc interval, and wide-complex tachycardia.

## Discussion

Our literature review has revealed that flecainide toxicity is frequently identified in clinical settings, where suspicion of intoxication can arise from clinical history or typical ECG-based manifestations [28, 29]. However, flecainide is rarely tested and even less commonly identified in forensic cases: clinical data and evidence of arrhythmias may not be available, and the deceased is often found at home. Flecainide intoxication can easily be suspected when a history of depression or evidence of overdose is discovered during the investigation, as commonly observed in fatal cases [10, 27, 32]. Nevertheless, intoxications can also occur in patients with cardiovascular disorders and abnormalities identified at postmortem examination [30, 31]. When a certain or highly probable cardiac substrate for sudden cardiac death is revealed at autopsy, toxicological analysis may not be pursued or may not include antiarrhythmic drugs, leading to erroneous interpretation.

In the present case, suspicion of flecainide toxicity arose from circumstantial data, together with primary and secondary death scene investigations, and was further supported by the absence of a definitive cause of death at autopsy. However, the amount of flecainide consumed remained uncertain, as the individual had self-prescribed two packages, but additional empty packages were found at his workplace. The potential role of coronary artery stenosis also required further clarification, necessitating a differential diagnosis between sudden cardiac death and flecainide intoxication.

Toxicological analyses detected flecainide in central and peripheral blood (3.411 mg/l and 2.962 mg/l, respectively), thus requiring careful interpretation.

It is known that the risk of flecainide-induced adverse effects increases at higher plasma or serum concentrations [57]. Our literature review, though, highlighted that flecainide concentrations can vary widely, with a large overlap between fatal and non-fatal concentrations. Particularly, serum levels up to 850–1940 mg/l were reported in non-fatal intoxications [49, 52].

The concentrations identified in our case were below the median values described for fatalities, as reported in our review (13 mg/l). This is particularly noteworthy given that the flecainide consumption was likely intentional. Levels were also below reported lethal concentrations, described as low as 2.6 mg/l, but exceeded the therapeutic range (0.2–1.0 mg/l) [57], suggesting potential toxicity. Recently, a case of sudden cardiac arrest due to flecainide, with a detected concentration of 1.9 mg/l was described [44]. The development of Takotsubo cardiomyopathy was also reported in an attempted suicide described by G. Bodziock et al. in which a serum flecainide level of 3.0 mg/l was detected two days after ingestion [45]. Although in both cases the fatal outcome was avoided, these reports further confirm that the concentrations of flecainide detected in our case could lead to toxicity.

A further issue in interpreting flecainide concentrations arises from the potential overestimation of postmortem levels. As reported in the literature, blood concentrations of flecainide might increase in postmortem samples compared with antemortem serum samples, with a reported ratio of approximately 3.6 [18, 58]. This postmortem increase was also demonstrated in a case reported by Yoshitome K. et al., in which antemortem concentrations were 2.5 mg/l and postmortem levels ranged between 13.8 and 44.2 mg/l [30].

A cardiac-to-peripheral blood concentration ratio (C/P ratio) greater than 1 is typically considered a sign of postmortem redistribution. In our case, the calculated C/P ratio was 1.15, suggesting a moderate tendency toward redistribution. This ratio is consistent with previous animal studies [18] and with a case of fatal intoxication included in our review (C/P ratio = 1.18) [27]. Moreover, given the relatively short interval between death and autopsy, and the tablet count at the scene, a significant increase in the postmortem concentrations seemed unlikely.

Considering the challenges in interpreting postmortem flecainide concentrations and the low blood drug concentrations despite intentional overdose, the impact of pharmacogenetic factors was additionally evaluated in the present case.

CYP2D6 is recognized as the primary enzyme for flecainide metabolism [59]. Unfortunately, in the present case, it was not possible to empirically assign star (*) alleles or haplotypes to the detected CYP2D6 variants.

Such assignments are typically based on genotyping tests that target only the most common and well-characterized sites of functional variation. However, previously uninterrogated sites of variations may also contribute to loss of function. Rs1065852 and rs1135840 are core variables of *10 allele, which has an activity value of 0.25 [60], and this may have had an influence in the present case.

Genetic variations in CYP3A5 can also influence the metabolism of flecainide [60]. Although the literature on the topic is limited, CYP3A5*3/*3 genotype has been associated with significantly higher systemic exposure and impaired clearance of flecainide as compared with CYP3A5 *1/*1 + *1/*3 [11, 61]. This suggests a PM phenotype, which may not have been clinically evident while therapeutic doses of the drug were used. However, when multiple doses of flecainide were consumed, this phenotype could have contributed to increased toxicity, even though blood levels remained relatively low.

Although genetic testing may not be necessary in more straightforward cases, this case highlights the importance of considering pharmacogenetic factors when interpreting flecainide toxicity. In addition to genetic variability, gender-related differences in toxicokinetics and toxicodynamics have been suggested as potential modifiers of overdose risk and outcomes in drug intoxications [62]. Although no clear gender predominance emerged in the present review, the potential contribution of gender-related factors warrants consideration in future investigations of flecainide toxicity.

Looking ahead, forensic toxicology will face increasing challenges in interpreting multi-analyte data in the context of individual genetic variability. Taking into account this perspective, collaborations between toxicologists, geneticists, and pathologists will be crucial to advance comprehensive and personalized forensic death investigation approaches [63]. Moreover, these data could be useful for comparisons in the clinical setting, where the focus is on optimizing medication dosing on patient-specific factors, including drug-metabolizing enzyme genotypes [64].

Among the cases reported in our literature review, pathological findings associated with flecainide intoxication were mostly unremarkable [31, 37–39]. Visceral congestion, pulmonary edema, as well as brain edema [9] were noted, and this is consistent with the findings of our case. Hepatic congestion and bilateral lung edema were also described in a case of suspected flecainide toxicity, although the cause of death was classified as natural [31]. This once again underlines the complexity of interpreting postmortem findings in similar cases.

Moreover, in our literature review, in a substantial proportion of fatal and non-fatal cases, flecainide was consumed in combination with psychotropic substances such as alcohol, benzodiazepines, or opioids, which is consistent with epidemiological data on self-poisoning and suicide attempts [65]. This pattern of polydrug use, not observed in our case, may additionally potentiate cardiotoxic effects and complicate interpretation of toxicological findings.

In the present case, no acute or chronic obstruction of the coronary arteries was found. Despite the young age and the absence of cardiovascular risk factors, mild-to-moderate coronary artery disease was detected at postmortem examination. This type of cardiac substrate, likely unknown before death, might have increased the risk of sudden death due to cardiac arrhythmias [66]. Moreover, cardiac histology allowed the identification of myofiber waviness with disarray, foci of coagulative necrosis of cardiomyocytes, and diffuse interstitial edema, which might represent signs of cardiogenic shock on a slightly compromised substrate.

However, the man was also resuscitated, and this might also explain the described findings.

Based on a comprehensive analysis of all available data, including circumstantial data, chemical-toxicological analyses, postmortem genetic test and literature data, the cause of death was determined to be intentional flecainide intoxication. In the context of a PM phenotype and moderate coronary artery disease, flecainide most likely caused a fatal arrhythmia and cardiogenic shock.

## Conclusions

In cases of sudden death among young adults prescribed flecainide, death often tresults from intentional flecainide intoxication, which is frequently confirmed by circumstantial data and death scene investigation.

However, pathological findings in flecainide intoxication are often non-specific, and cardiovascular abnormalities might suggest a natural cause of death. The interpretation of toxicological results remains challenging due to the overlap in flecainide concentrations between fatal and non-fatal intoxications, as well as the potential for postmortem increases in flecainide levels. Further research is warranted to better understand the impact of postmortem changes on blood levels and the influence of genetic variants of drug-metabolizing enzymes.

## Key points

•Flecainide toxicity could arise even at slightly elevated blood levels.

•Postmortem concentration could be affected by artifacts.

•Pharmacogenetic testing could provide insights for forensic interpretation.

## Supplementary Information

Below is the link to the electronic supplementary material.

Supplementary file1 (DOCX 47 KB)
