# Supplementary material for: Suicide by flecainide: a case report and review of the literature
Source: Forensic Sci Med Pathol. 2026 Mar 7;22(3):944–56. doi: 10.1007/s12024-026-01188-8 (PMC13672392; doi:10.1007/s12024-026-01188-8)
Supplement: Supplementary file 1 [file 12024_2026_1188_MOESM1_ESM.docx]

**Supplementary material to: Suicide by flecainide: a case report and review of the literature**

The following sections are included in the Supplemental material.

A. *Materials and methods. Details on validation of method – Table A*
B. *Materials and methods. Details on pharmacogenetics testing*
C. *Results of pharmacogenetics testing – Table C*
D. *Results of literature review – Table D*
A. *Materials and methods. Details on validation of method*

The validation of the method included: limit of detection (LOD), lower limit of quantification (LLOQ), linearity, analytical bias, carry-over, matrix effect (ME), recovery (RE), and dilution integrity of the method were previously evaluated throughout the method validation process in accordance with suggestions made by the Organization of Scientific Area Committees (OSAC) for Forensic Science [1], with calibration curves performed over five consecutive days. Validation parameters of the HPLC-MS/MS method for the quantification of flecainide, showing linearity, limit of detection (LOD), lower limit of quantification (LLOQ), precision and accuracy are shown in Table A.

Table A. Validation parameters of the HPLC-MS/MS method for the quantification of flecainide, showing linearity, limit of detection (LOD), lower limit of quantification (LLOQ), precision and accuracy.

Occorre anche riportare detailed precision and accuracy results rather than just ranges. E anche carry-over, matrix effect recovery, dilution integrity.

**Table A**

| **Analytes of interest** | **Calibration range** | | **n. calibration point** | **R^2^ and Mandel test** | **weighing** |
| --- | --- | --- | --- | --- | --- |
| **Flecainide** | 0.5-1000 ng/ml | | 8-points calibration curve | R^2^= 0.995  Mandel test coefficients (p value and Fcrit 95%)[2] | 1/x^2^ weighting |
|  | **LOD**  **[ng/mg]** | **LLOQ**  **[ng/mg]** | **Precision (CV%)**  **Intraday (n=3) Interday (n=5)** | **Accuracy (bias%)**  **Intraday (n=3) Interday (n=5)** |  |
|  | 0.17 ng/ml | 0.5 ng/ml | within ± 15% | within ± 15% |  |

Linearity as tested by five calibration curves performed in five different days.

Precision (coefficient of variation or CV%) = 100 x standard deviation/mean.

Accuracy (bias%) = 100 x [(mean concentration found – nominal concentration)/nominal concentration].

LOD = defined by signal to noise 1:3 with respect to LLOQ.

LLOQ = accuracy and precision were within ± 20%

References

[1]	K. Yang, Organization of Scientific Area Committees (OSAC) for Forensic Science Standard Practices for Method Development., NIST (2016). https://www.nist.gov/organization-scientific-area-committees-forensic-science (accessed March 2, 2024).

[2]	H. Ahrens, Mandel, John: The Statistical Analysis of Experimental Data. Wiley & Sons, Inc., New York 1964, 53 Abb., 109 Tab., 7 Taf., Biom. Z. 10 (1968) 274–275. https://doi.org/10.1002/bimj.19680100407.

B. *Materials and methods. Details on pharmacogenetics testing*

Libraries were prepared according to the Ion AmpliSeq™ Library Kit 2.0 protocol (Life Technologies, Thermo Fisher Scientific, MAN0006735 rev C.0). Libraries enrichment and chip loading steps was performed by using the Ion Chef™ (TFS) and the Ion 520™ Kit. Sequencing was performed on Ion Gene Studio S5^TM^ (TFS). All raw data was processed by the Torrent Suite (v. 5.12.3) and the reads aligned against human reference genome (GRCh37/hg19). Coverage analysis was carried out by Coverage Analysis (v5.12.0.0) plugin, which provided information about mapped reads, on-target percentage and mean depth of coverage downloadable for each sample library. Allelic call for each library were performed through variantCaller Plugin (v.5.12.0.4). The software Integrative Genomics Viewer (IGV) was used to verify the call variants.

Haplotype translation files were created using data from PharmGKB and used in conjunction with AlleleTyper™ Software (ThermoFisher Scientific) to determines the star-allele results.

C. *. Results of pharmacogenetics testing.*

Sequencing generated 261510 reads (aligned reads 261190, mean read length 132 bp). The average base coverage depth was 1741 (uniformity of base coverage 99.99%) and all target bases had a coverage of at least 30X. Copy number variations (CNVs) in CYP2D6 were not investigated.

**Table C**. CYP2D6 genotype and reference as reported in pharmvar [65]

| CYP 2D6 | rs1135840 | rs28371730 | rs2004511 | rs59421388 | rs28371725 | rs5030867 | rs16947 | rs5030656 | rs35742686 | rs2267447 | rs111564371 | rs112568578 | rs113889384 | rs72549354 | rs3892097 | rs5030865 | rs5030655 | rs1058164 | rs61736512 | rs1081003 | rs28371706 | rs201377835 | rs72549357 | rs5030862 | rs1065852 | rs769258 | rs1080985 |
| --- | --- | --- | --- | --- | --- | --- | --- | --- | --- | --- | --- | --- | --- | --- | --- | --- | --- | --- | --- | --- | --- | --- | --- | --- | --- | --- | --- |
|  | G/  C | C/C | T/C | C/C | C/C | T/T | G/G | T/T | T/T | T/C | T/C | C/G | G/A | -/- | C/C | C/C | A/A | G/C | C/C | G/A | G/G | C/C | -/- | C/C | G/A | C/C | G/G |
| Ref. **1* | C | C | T | C | C | T | G | TCT | T | T | T | C | G | - | C | C | A | C | C | G | G | C |  | C | G | C | G |

D. *Results of literature review.*

**Table D**. Cases of non-fatal acute intoxication related to flecainide

| **Gender, Age**  **(y-o)** | **Flecanide concentrations (mg/l or mg/kg)** | **Other drugs detected in blood (mg/l or mg/kg)** | **Manner of intoxication** | **Main clinical findings** | **Ref.** |
| --- | --- | --- | --- | --- | --- |
| M, 72 | Serum  - 3 h after ingestion 2.8  -27 h: 2.1  -32/44 h: 1.9  -56 h: 1.6  Urine  from 3 h to 56 h :> 5 | - | Intentional flecainide intoxication | History of coronary artery disease, sick sinus syndrome with a pacemaker, and ischemic cardiomyopathy. Loss of consciousness, hypotension, tachycardia, QRS prolongation | McCabe DJ. et al 2022  [34] |
| M, 47 | Blood  Flecainide: 2.98 | Amiodarone 4.8 | Intentional flecainide and amiodarone intoxication | History of depression and atrial fibrillation. ED symptoms: fatigue, anxiety, headache, GCS 12. The patient developed cardiotoxicity, with PR prolongation, AV block, widened QRS | Bologa C. et al. 2021  [35] |
| 3 months | Serum: 1.2 | - | Accidental flecainide intoxication | Drowsiness, lack of appetite, prolonged AV interval, QRS widening, acute intra-atrial re-entrant tachycardia | Correra A. et al. 2021  [36] |
| M, 86 | Serum/plasma: 2.44 | - | Accidental flecainide intoxication | Heart failure in a patient with pacemaker, wide QRS | Rivner H. et al.  2021  [37] |
| Case1) F, 3 days  Case 2) M, 2 months | Case 1) Serum: 0.72  Case 2) Serum: 1.1 | - | -  Flecainide intoxication | Case 1) History of SVT. Brought to the hospital with dyspnea after her last flecainide dose. She developed sinus rhythm with a prolonged PR interval, complete right bundle branch and a prolonged QTc interval  Case 2) History of SVT. ECG revealed sinus bradycardia with prolonged PR interval, complete right bundle branch and a prolonged QTc interval | Vaquer G. et al. 2020  [38] |
| M, 57 | Serum/plasma: 1.99 | - | Accidental flecainide intoxication | History of HIV, end stage renal disease, atrial flutter. Admitted for lower extremity weakness, developed wide complex tachycardia and ventricular fibrillation | Drutel R. et al. 2020  [39] |
| M, 48 | Serum: 3.03 | Urine: benzodiazepine and opiates | Intentional flecainide intoxication | History of PE with embolectomy 3 years prior, and paroxysmal atrial fibrillation treated with flecainide | Bodziock G. et al. 2019  [40] |
| F, 68 | Serum: 2.44 | Bisoprolol | Flecainide intoxication | History of atrial fibrillation, sick sinus syndrome, followed by pacemaker implantation, and chronic renal failure. Home medications were bisoprolol bumetadine, flecainide, and rivaroxaban. Brought to the hospital after collapsing. ECG showed extreme broad QRS complexes and loss of pacemaker capture. After sodium bicarbonate and intravenous lipid emulsion treatments, the patient was discharged | Heldens M. et al. 2019  [41] |
| F, 86 | Serum: 1.39 | - | -  Flecainide intoxication | History of atrial fibrillation and pacemaker implantation. Home medication were flecainide and anticoagulants. Brought to the ED with abdominal pain, weakness, nausea and dry heaves. ECG revealed a wide complex rhythm with pacemaker spikes. Treated with bicarbonate IV | Apfelbaum J. et al. 2018  [42] |
| M, 23 | Serum: 2 | Amphetamine | Intentional flecainide intoxication | History of symptomatic premature atrial complexes, paroxysmal atrial tachycardia, and severe depression. Developed a wide complex rhythm degenerated to pulseless ventricular tachycardia and ventricular fibrillation | Vu NM. et al. 2016  [43] |
| M, 46 | Serum: 1.76 | - | Intentional flecainide intoxication | History of surgically treated patent foramen ovale, and symptomatic PVCs for which he was recently started on flecainide 50 mg every 12 h. ED: dizziness, nausea and lethargy, wide complex tachycardia | Valentino MA. et al. 2016  [15] |
| F, 13 | Serum:  After 90 m: 1.512  After 9 h: 2.699  After 12 h: 2.075  After 24 h: 0.168  After 60 h: 0.895  After 140 h: <0.100 | Bisoprolol and aspirin | Intentional flecainide intoxication | The patient had a first-degree AV block, prolonged QTc, VF arrest, and Brugada-like syndrome | Mukhtar O. et al. 2015  [44] |
| F, 24 | Serum 11.085 | Levetiracetam | Intentional flecainide intoxication | Brought to the ED in profound shock with a wide complex bradydysrhythmia. She developed pulseless electrical activity cardiac arrest. After venoarterial extracorporeal membrane oxygenation, she discharged home on day 19 | Reynolds et al.  2015  [45] |
| F, 52 | Serum: 4.13 | - | -  Flecainide intoxication | History of paroxysmal atrial fibrillation, Gitelman syndrome and depression. She was found unresponsive at home because of a cardiac arrest. ECG showed a wide QRS complex and profound bradycardia. After bicarbonate, intravenous fat emulsion and activating charcoal the patient was discharge | Sivalingam SK. et al.  2013  [46] |
| M, 18 days | Serum: 1.36 | - | Accidental flecainide intoxication | Past medical history of SVT in therapy whit flecainide acetate 4 mg every 8 h. Developed lethargy, bradycardia, absence of pulse, wide-complex tachycardia | Jang DH. et al.  2013  [47] |
| M, 51 | Serum:  After 1 h: 1.8  After 8.5 h: 2.76 | - | Intentional flecainide intoxication | History of atrial fibrillation and hypertension. Home medications were alprazolam, flecainide, lisinopril and aspirin. ECG demonstrated a wide QRS complex, prolonged QTc, bradycardia and first-degree AV block | Ellsworth SH. et al. 2013  [48] |
| F, 57 | Serum: 1940 | - | Intentional flecainide intoxication | History of supraventricular tachyarrhythmia and chronic therapy with flecainide. Admitted to ED with wide QRS complex and prolonged QTc interval. Treated with sodium bicarbonate | Rognoni A. et al. 2009  [49] |
| F, 34 | Serum: 3.6 | - | Intentional flecainide intoxication | History of paroxysmal SVT. The patient took an intentional dose of 4500 mg of flecainide. She showed irregular wide complex rhythm | Devin R. et al.  2007  [50] |
| F, 47 | Serum: 2.34 | Amitriptyline, losartan, and amlodipine | - | History of arterial hypertension and paroxysmal atrial fibrillation. The patient had wide QRS complex and sine wave appearance | Timperley J. et al. 2005  [51] |
| F, 45 | Serum: 850 | Alcohol | Intentional flecainide and alcohol intoxication | Brought to the hospital after ingestion of 2000 mg of flecainide and 18 g of alcohol. On admission, she was conscious and responsive. Later she developed therapy-resistant ventricular fibrillation with cardiopulmonary arrest. Intravenous administration of amiodarone for 72 h. She recovered completely after 5 days | Siegers A. et al. 2001  [52] |
| F, 20 | Serum: >4.0 | Paracetamol | Intentional flecainide intoxication | History of paroxysmal SVT and depression. Brought to the ED after intentional ingestion of 4000 mg of flecainide. She manifested wide complex rhythm. Successfully treated with cardiopulmonary bypass | Corkeron MA. et al.  1999  [53] |
| F, 15 | Serum: 5 | - | Intentional flecainide intoxication | ECG showed a wide QRS irregular bradycardia, and prolonged QTc interval. She developed an atrioventricular block. | Hanley NA. et al.  1998  [54] |
| Case 1)  F, 38  Case 2)  M, 61 | Case 1)  Plasma/serum: 2.18  Case 2)  Plasma/serum 3.0 | - | Intentional flecainide intoxication | Case 1) history of depression. Agitated, vomiting, dizziness, hypotension, wide QRS  Case 2) history of atrial fibrillation. After ingestion of flecainide and unknown quantities of losartan and ranitidine, lethargy wide QRS | Lovecchio F. et al. 1998  [55] |
| F, 28 | Serum:  2h: 3.7  4.5h: 2.5  8h: 1.75  19h: 1.15  35h: 0.7  59h: 0.4 | Alcohol, diazepam, loperamide | Intentional flecainide intoxication | Brought to the hospital somnolent with mydriatic pupils, and shallow breathing respiration. Developed polymorphous ventricular tachycardia. She was intubated and placed on mechanical ventilation. A gastric lavage was performed | Winkelmann BR. et al. 1987  [56] |
| AV, atrioventricular; ECG, electrocardiogram; ED, emergency department; F, female; M, male; GCS: Glasgow coma scale; SVT, supraventricular tachycardia; QTc, heart-rate corrected QT interval. | | | | | |
